# Coding complete sequence of three segments of Allium deltapartitivirus from Iraq

**DOI:** 10.1128/mra.01108-23

**Published:** 2024-01-18

**Authors:** Amer Muhsen, Osamah Alisawi, Maadh Al Fahad

**Affiliations:** 1 Plant Protection Department, Faculty of Agriculture, University of Tikrit, Salaadin, Iraq; 2 Plant Protection Department, Faculty of Agriculture, University of Kufa, Najaf, Iraq; Katholieke Universiteit Leuven, Belgium

**Keywords:** RNA seq, bioinformatics, onion genome, Allium deltapartitivirus

## Abstract

Here, we report the coding complete sequence of three segments of the Iraqi isolate of Allium deltapartitivirus named Tikrit in onion plants. According to the phylogeny, this isolate is closely related to an Allium deltapartitivirus from Brazil and to Arhar cryptic virus 1 from Hyderabad.

## ANNOUNCEMENT

**Fig 1:**
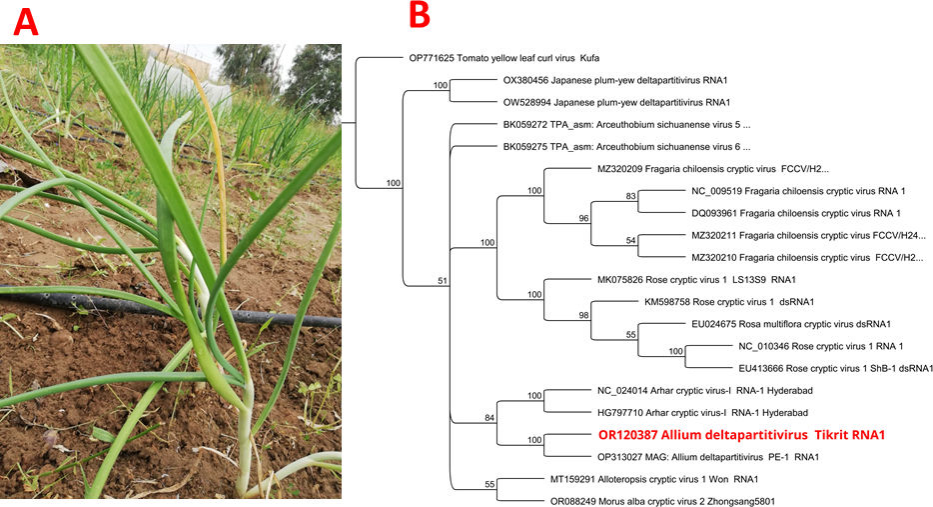
Onion plant in Tikrit region shows slight chlorotic symptoms where leaf samples were taken (**A**). The phylogeny tree for Allium deltapartitivirus segment RNA1 shows close relationship to OP313027 isolate of deltacryptic virus identified in *Allium cepa* from Brazil (2) with pairwise identity: 95.0%. The trees were built by Geneious tree builder v. 2023.2.1, and the alignments of full-genome nucleotide sequences were performed with ClustalW (6). A maximum likelihood was constructed using the best substitution model Hasegawa-Kishino-Yano (HKY), and the tree was inferred with 500 bootstraps. The out-group member was *tomato yellow leaf curl virus* (**B**).

The Partitiviridae family comprises encapsidated, bisegmented dsRNA viruses that can infect plants, fungi, or protozoa. The taxonomy of this family has been changed, and the former genera *Partitivirus*, *Alphacryptovirus*, and *Betacryptovirus*, have been replaced with genera *Alphapartitivirus*, *Betapartitivirus*, *Gammapartitivirus*, and *Deltapartitivirus*. A metagenomic approach and the mining of sequence databases have also been used to assemble novel partitivirus sequences and find evidence for partitivirus-host interactions and host effects (1). Recently, Cruz and colleagues used RNA-seq data derived from onion tissue to assemble the genome of a novel partitivirus composed of three dsRNAs, which were closely related to the Arhar cryptic virus 1, which was constructed from *Allium cepa* samples. In the Partitiviridae family, the virus is classified under the genus *Deltapartitivirus* with the suggested name Allium deltapartitivirus (2). For the current study, leaves were taken from symptomatic onion plant in Tikrit region, Saladin Province on 3 February 2023 (Fig. 1A) and carefully cut into square pieces measuring 0.5 × 0.5 cm. Subsequently, each square piece was immersed in 5× volume RNAlater (2 mL) in a single Eppendorf tube. As replicates, five samples were sent to DNA Link in the Republic of Korea, and only one sample was processed. The RNeasy Plant Mini Kit (QIAGEN, Hilden, Germany) was used to extract RNA from plant samples according to instructions. A total RNA Library Prep Kit from TruSeq was used to prepare the sequencing library, and it was subjected to whole-genome sequencing (Platform: Novaseq6000; Application: WTS/mRNA), and the reads were then trimmed using Trimmomatic-0.39 and BBduk v 37.22 in Geneious Prime 2023.2.1 software (3). As a result, 98,945,112 paired-end reads of 101 bases were obtained from the RNAseq library. An NCBI-GenBank database of 5,040 virus sequences has been constructed to provide a reference genome database (https://bitbucket.org/osamahalisawi/workspace/projects/OV). For mapping the clean and paired-end reads against the reference genome database, Geneious Prime was used. Geneious RNA mapper was used to map RNASeq data against the reference genome database (sensitivity: medium-low sensitivity). This mapping analysis showed 71,913, 238,379, and 231,169 reads mapped to Allium deltapartitivirus segments RNA 1, 2, and 3 (OP313027, OP313028, and OP313029), respectively. The consensus sequences of the three segments were 1,728, 1,466, and 1,425 bp, respectively. The coverage depths were 21,454, 38,345, and 64,536, respectively. The sequences were extracted, aligned with the reference genome, and then annotated using the Open Reading Frame Finder tool (NCBI’s version: https://www.ncbi.nlm.nih.gov/orffinder/) (4) and BLASTx v.5 (5). All tools were run with default parameters, unless otherwise specified. The RNA1 encodes RNA-dependent RNA polymerase, and the RNA2 and RNA3 encode coat protein 1 and coat protein 2, respectively. The phylogeny confirmed the close relationship between the Iraqi and Brazilian isolates and also the Arhar cryptic virus 1 from Hyderabad (Fig. 1B). The diagnosis of the coding complete sequence of the Allium deltapartitivirus is the second registration of this emerged virus.

## Data Availability

This whole-genome shotgun project has been deposited in GenBank under the accession no. SRR26142870. The version described in this paper is the first version. The coding complete sequence of Allium deltapartitivirus segments has been deposited in GenBank under accession numbers RNA1 (OR120387), RNA2 (OR120388), and RNA3 (OR120389).
